# Pharmacy services and psychiatric patient satisfaction among community-based health insurance users in Ethiopia

**DOI:** 10.3389/fmed.2024.1469832

**Published:** 2024-11-07

**Authors:** Gashaw Sisay Chanie, Wagaye Atalay, Tekletsadik Tekleslassie Alemayehu, Zemenu Wube Bayleyegn, Gebresilassie Tadesse, Setegn Fentahun, Yilkal Abebaw Wassie, Tegenu Chanie Tesfaye, Gebremariam Wulie Geremew

**Affiliations:** ^1^Department of Clinical Pharmacy, School of Pharmacy, College of Medicine and Health Sciences, University of Gondar, Gondar, Ethiopia; ^2^Department of Social and Administrative Pharmacy, School of Pharmacy, College of Medicine and Health Sciences, University of Gondar, Gondar, Ethiopia; ^3^Department of Psychiatry, School of Medicine, College of Medicine and Health Sciences, University of Gondar, Gondar, Ethiopia; ^4^Department of Medical Nursing, School of Nursing, College of Medicine and Health Sciences, University of Gondar, Gondar, Ethiopia; ^5^Department of Pharmacy, Debre Markos University, Debre Markos, Ethiopia

**Keywords:** community-based health insurance, patient satisfaction, pharmacy service, psychiatric patients, Ethiopia

## Abstract

**Background:**

The development of community-based health insurance (CBHI) was driven by the need to provide economic protection for the poor against unexpected healthcare expenses. This can lead to increased patient satisfaction with their overall care. Maintaining high levels of client satisfaction with pharmacy services is crucial for effectively treating patients with psychiatric disorders. Therefore, the purpose of this study was to assess the impact of pharmacy services on psychiatry patient satisfaction among users of CBHI in the psychiatric setting.

**Methods:**

A multicenter cross-sectional study was conducted at the psychiatric clinics with a systematic random sampling technique between December 2023 and May 2024. A structured interview questionnaire was used to gather data. Both bivariate and multivariate analysis were employed. In multivariate analysis, variables having a *p*-value of < 0.05 were deemed statistically significant.

**Result:**

A total of 420 participants were enrolled with a response rate of 99.5%. Overall, 261 (62.1%) of the patients reported being satisfied with the pharmacy service. However, a significant number of patients expressed dissatisfaction with certain aspects of the service, such as pharmacist counseling on medication side effects (43.1%), medication interactions (36.9%), and labeling and dispensing of medicines (42.4%). According to the study, living in an urban area [AOR = 2.0; 95% CI (1.25, 3.2); *P* = 0.04], being between the ages of 18–35 and 36–44 [AOR = 2.7, 95% CI (1.38, 5.3), *p* = 0.04] and [AOR = 3.3, 95% CI (1.6, 5.7), *p* = 0.001] respectively. First and second visits to the institution [AOR = 2.2; 95% CI (1.15, 4.4); *P* = 0.01] and [AOR = 1.9; 95% CI (1.13, 3.3); *P* = 0.01] respectively. Having two psychiatric disorders [AOR = 1.8, 95% CI (1.07, 3.07), *p* = 0.02] and all drug availability [AOR = 1.5, 95% CI (1.3, 3.43), *p* = 0.02], were positively associated with psychiatry patient pharmacy service satisfaction.

**Conclusion:**

In this study, the users of CBHI psychiatric patients were generally satisfied with the pharmacy service. Additionally, being urban residency age (18–44 years), first and second visits to the institution, having two psychiatric disorders and all drug availability were found to have a significant impact on psychiatric patient pharmacy services satisfaction.

## Introduction

Community-based health insurance (CBHI) is defined as the aspiration that all people receive the quality health services they need without suffering financially as a result of seeking healthcare (1). All around the world, CBHI programs have been put into place. The need for the poor to have economic defense against calamity healthcare expenses resulted in the development of the CBHI. In addition to creating more income for the healthcare industry, CBHI intends to lower out-of-pocket costs and broaden access to healthcare services (2).

Millions of individuals worldwide suffer and even lose their lives due to the inability to afford medical care. The lack of sufficient funding for healthcare services prevents any nation from providing comprehensive health coverage to its entire population. According to the World Health Organization (WHO), 150 million people experience financial hardship each year, while out-of-pocket payments for healthcare services push over 100 million individuals into poverty. In the United States alone, reports show that 46 million people lack adequate health insurance, highlighting that even one of the world's wealthiest nations struggles to offer full coverage to all its citizens (3, 4).

Most healthcare services in Sub-Saharan Africa are paid for out of pocket by the patient, which frequently results in the usage of inadequate medical care. But a risk-pooling healthcare system such as the CBHI scheme has lately come to light as a viable substitute. This should lead to increased use of healthcare services, fewer income shocks from illness, and eventually an affordable and fully operational global medical care system (5). The difficulties with higher prices, confidence in the managers' competency and honesty, the benefits package's attraction, and the providers' capacity to deliver high-quality care (6).

Health insurance coverage in low-income and middle-income countries: progress made to date and related changes in private and public health expenditure (7). Healthcare professionals are concerned about the rising expense of healthcare and the requirement to make better use of the resources at their disposal. Particularly in low- and middle-income nations, there is constant research being done on various methods of funding health care systems. This is because their health systems consistently receive little funding (8, 9).

The goal of psychiatric therapy is to enhance social and daily functioning while maintaining high care standards. Patient satisfaction is crucial in managing chronic conditions and is a key indicator of care quality (10). Factors like inadequate facilities, overworked staff, medication shortages, stigma, long wait times, treatment type, and service cost impact satisfaction (11–13). Various studies have also demonstrated that patient satisfaction is influenced by a multitude of factors, including patient demographics (14, 15), treatment program (16), diagnosis and duration of disease (17, 18), and patient expectations (19), in addition to the quality of service delivery.

One component of healthcare that must be provided to the highest standards in order to raise the standard of care provided overall is hospital pharmacy services (20). A hospital pharmacy unit's services are an essential part of the institutional health care system. The primary services provided by this unit include drug distribution and dispensing, compounding, medication use review, adverse drug reaction monitoring, and drug information service (21). In this hospital pharmacy service medication unavailability, busy pharmacy staff, poor design of dispensing area, and cost of medication specifically affect patient satisfaction in addition to the above factors (11, 12, 22). Positive service quality is anticipated to be positively impacted by successful pharmacist performance, which may be indicated by high patient satisfaction (23–25).

Utilizing only mental symptoms as a proxy for level of care since contentment can be a relative term, it's crucial to find out how happy patients are with the care they receive (26). Patient satisfaction is a key performance indicator for mental health services, yet limited data exists on psychiatric patients' satisfaction with pharmacy services in Ethiopia's referral hospitals. Pharmacy services significantly impact patient satisfaction and hospital reputation, but staff often struggle to meet patient and societal needs. Regular surveys are essential to assess and improve pharmacy care in psychiatric settings (27). Over the past 30 years, mental health services have been offered in the most part of the country, and patient service satisfaction is a trustworthy sign of high-quality medical care (28). While a large number of researches on patient satisfaction have been carried out in low- and middle-income nations, relatively few have been carried out in Ethiopia. Moreover, no studies have been conducted in this particular area. Therefore, the purpose of this study was to determine the influence of pharmacy service on psychiatric patient satisfaction among community-based health insurance users for psychiatric patients.

## Method and materials

### Study design, period, and setting

CBHI-implementing districts in the Amhara region of Ethiopia, adult psychiatric patients from December 2023 to May 2024, were included in this multicenter cross-sectional study. The investigation was carried out in Northwest Ethiopia at tertiary care hospitals' psychiatric follow-up clinics. These facilities were chosen using a lottery process from among nearby institutions. The Felege Hiwot Comprehensive Specialized Hospital Psychiatric Clinic (FHCSH), the University of Gondar Comprehensive Specialized Hospital Psychiatric Clinic (UoGCSH), and the Tibebe-Ghion Comprehensive Specialized Hospital Psychiatric Clinic (TGCSH) were the hospitals that were part of the study. The UoGCSH is located 750 kilometers and 560 kilometers from Addis Ababa and is the location of both FHCSH and TGCSH (29).

### Study participants

The source population comprised all CBHI users' adult psychiatry patients in the designated hospitals' CBHI implementation zones and all CBHI scheme users' adult psychiatry patients found at the selected psychiatric clinic hospitals during the study period were considered as the study population.

### Inclusion and exclusion criteria

Psychiatric patients older than 18 years who were enrolled in the CBHI scheme and who had received pharmacy services from the CBHI contracted institution within the previous 6 months were included. Patients who were incapable of responding, had cognitive impairment, communication or hearing issues, or were in critical condition were excluded.

### Sample size determination and sampling techniques

Utilizing a single population formula, *n* = (Zα/2)2 p(1-p)/d2, we determined the sample size by assuming the following: d = a margin of error of 0.05, a non-response rate of 10%, Za/2 = 1.96 (at a two-sided 95% CI), and *p* = 50.3% from a prior work (30).


$$\begin{array}{l} n=\frac{{\left(z_{\frac{a}{2}}\right)}^{2}\times p\times \left(1-p\right)}{d^{2}}=\frac{{\left(1.96\right)}^{2}\times 0.503\times \left(1-0.503\right)}{{\left(0.05\right)}^{2}}\;+10\%\; \end{array}$$

*n* = 422 was determined as the sample size.

It was then distributed proportionately among the chosen hospitals to make the sample representative of the study population. As a result, UoGCSH, FHCSH, and TGCSH numbered 141, 141, and 140, respectively. Considering the number of individuals who suffer from psychiatric disorders found in hospital records before the study's commencement, a proportionate allocation was created.

A systematic random sampling technique was used to approach individuals from chosen institutions using their unique medical identity numbers. Initially, a simple random sampling method was utilized to select the starting sample. Then, until the target sample size was reached, eligible participants were added to the research based on a sampling interval.

### Data collection tool and quality assurance

A structured interview questionnaire that was created following a thorough analysis of relevant literature on the subject was used to gather data (31, 32). The tool had two components: part one sociodemographic profile, including gender, age, residence, marital status, educational status, and occupation. Part two had subheadings that were used to organize patient satisfaction. First, questions concerning participants' experiences with pharmaceutical services were asked. In the second subheading, participants were asked about their opinions about the pharmacy's environment, the availability of medications, and the price; under the third and fourth subheadings, participants were asked about their satisfaction with the pharmacist's approach or communication, and medication instructions were used to assess the outpatient services they received overall during their most recent visit. Patients were asked to score their level of satisfaction on a five-point Likert scale in all of the satisfaction questions (1: very satisfied, 2: satisfied, 3: neutral, 4: dissatisfied, and 5: very dissatisfied). Next, the satisfaction score was split into two groups: those who were satisfied ( ≤ 30) and those who were not satisfied higher than 30 (32).

To maintain consistency, it was initially prepared in English, and then translated into Amharic, then back to English. Data collection was done using the Amharic version. The instrument was used to gather information on sociodemographics, comorbid conditions, satisfaction with pharmacy service, variables relevant to the CBHI system, and other items (33). Senior pharmacy professionals and qualified nurses, respectively, handled the supervision and data collection. Supervisors and data collectors received 2 days of training on the goals, techniques, and protocols of the data-gathering process to guarantee the quality of the data. One week before the primary data collecting period, a pretest of the tool was created using 5% of the entire sample to verify the questions' coherence, clarity, and order as well as to acquaint participants with it. Every day, principal investigators and supervisors verified the accuracy, consistency, and completeness; following this, minor adjustments were made. A group of experts in the domains of epidemiology, biostatistics, public health, and pharmacy assessed the content validity. A reliable indication was included in the final edition of the questionnaire, which was encouraging (as demonstrated by the Alpha Cronbach test result of 0.822).

### Data analysis

Epi-data version 3.1 and SPSS version 27 were used for data entry and analysis, respectively. Tables and figures were used to convey the descriptive statistics that were collected. With binary logistic regression, the hyperlink between the components and outcome variables was ascertained. The outcomes of logistic regression stated that the selected model was a good logistic regression model fit, since the hosemer-lemeshow goodness of fit *p-*value is > 0.05 (patient satisfaction; *P* = 0.38), then fails to reject the null hypothesis and it is stated that the logistic model is good fit for the data set. Assumptions like outliers were checked before running the analysis and the data values had no outliers (cooks distance test 0.065 which was between 0 and 1). We took into consideration Cronbach's alpha coefficient to ensure the validity of the instruments employed. We included all variables with *p* < 0.25 in the bivariate analysis in the multivariate analysis to compensate for any potential confounders. To verify normality, both graphical and numerical techniques were applied. The data point in a Q-Q plot test was close to the oblique line, suggesting a normally distributed set of data. A bell-curve appearance in the histogram suggested that the data came from a standard population. Assumption of multi-collinearity was met as variance inflation factor is < 10 and tolerance >0.1 for all variables. To find independent determinants of patient satisfaction with the pharmacy service among CBHI users, the adjusted odds ratio with a 95% CI and a *p* < 0.05 was employed. Duration of disease, number of psychiatric disorder, marital status, educational level, and age were among the potential confounding factors that were taken into account by multivariate analysis and stratification in our primary analyses.

## Operational definition

Community-based health insurance scheme (CMHI) is health insurance that pools members' premium payments into a collective fund, which is managed by the members and covers basic healthcare costs at local health centers when a member is sick (34).

Patient satisfaction is a measure of the extent to which a patient is content with the health care they receive from their health care provider (35).

## Ethical consideration

Ethical clearance was obtained from the School of Pharmacy Research Review Committee on behalf of the University of Gondar research and ethical review board (SOP/325/2023). We obtained participants' informed written consent for the study. Confidentiality was ensured through no patient's name and identifier was taken rather codes were used as identifiers. Information regarding patients was used for the study purpose only.

## Results

### Socio-demographic characteristics of the study population

Out of 422 psychiatric patients approached, 420 patients agreed to participate, giving a response rate of 99.5%. Of them, 117 (27.7%) were aged 45–55 years, and the mean (±SD) age of respondents was 45 (±13.3) years. More than half of the patients 267 (63.3%) were urban residents. About 184 (43.8%) visited the health institution two times in their follow-up. Half of 214 (51.0%) of the patients who had two psychiatric disorders. Less than half of 176 (41.9%) study patients access some availability of medicine in the institution during their follow-up time (Table 1).

**Table 1:** Socio-demographic characteristics of the study participants, 2024 (*N* = 420).

**Socio-demographic variables**	**Categories**	**Frequency (%)**
Sex	Male	211 (50.2)
	Female	209 (49.8)
Age	18–35years	89 (21.2)
	36–44years	111 (26.3)
	45–55 years	117 (27.7)
	≥56years	103 (24.4)
Residence	Urban	267 (63.3)
	Rural	153 (36.7)
	Religious	Orthodox	187 (44.3)
Muslim		178 (42.2)
Catholic and protestant		54 (12.8)
Marital status	Single	80 (19)
	Married	248 (58.8)
	Divorced	69 (16.4)
	Widowed	23 (5.5)
Educational status	Unable to read or write	125 (29.6)
	Primary	106 (25.1)
	Secondary	99 (23.5)
	College and above	90 (21.3)
Employment	Unemployed	145 (34.4)
	Private employer	170 (40.3)
	Government employer	105 (24.9)
Familiarity to institution	First Visit	103 (24.6)
	Second visit	184 (43.8)
	More than 3 times	133 (31.6)
Psychiatric disorders	One	79 (18.8)
	Two	214 (51.0)
	Three and more	127 (30.2)
Duration of disease	6–12 months	138 (32.9)
	13–24 months	154 (36.7)
	>24 months	128 (30.4)
Availability of medicine	All available	148 (35.2)
	Available some	176 (41.9)
	Not available	96 (22.9)
Waiting time to service	< 15 min	144 (34.3)
	>15 min	276 (65.7)

### Psychiatric patient satisfaction with pharmacy service on the community-based health insurance scheme

Overall, approximately more than half (62.1%) of the patients were satisfied with the community-based health insurance (CBHI) scheme during Pharmacy Service delivery. Among the psychiatric patient CBHI users, more than half (51.6%) of the participants were satisfied and or very satisfied regarding the location of the pharmacy concerning other healthcare services, and the availability of prescribed drugs (51.9%). However, nearly half of the participants were unsatisfied and/or extremely dissatisfied with the healthcare providers' respectful care (49.5%), the pharmacy waiting area cleanness (41.9%), getting services in a short waiting time (41.9%), and labeling and dispensing medicines (42.4%) (Table 2).

**Table 2:** Level and overall psychiatric patient satisfaction with pharmacy service on the CBHI scheme (*N* = 420).

**Items**	**Satisfaction level** ***n*** **(%)**				
	**Very satisfied**	**Satisfied**	**Neutral**	**Dissatisfied**	**Very dissatisfied**
**The structural aspect of the setting**					
The location of the pharmacy is comfortable	123 (29.3)	166 (39.5)	28 (6.7)	65 (15.5)	36 (8.6)
Pharmacy location related to other healthcare service	106 (25.1)	112 (26.5)	79 (18.7)	65 (15.4)	56 (13.3)
The waiting area is clean and comfortable	78 (18.5)	104 (24.6)	60 (14.2)	90 (21.3)	87 (20.6)
Cleanness of pharmacy	91 (21.7)	63 (15.0)	114 (27.1)	79 (18.8)	71 (16.9)
Convenience of the dispensing area	121 (28.8)	138 (32.9)	44 (10.5)	75 (17.9)	41 (9.8)
**Medication availability and supply**					
Prescribed drugs availability	78 (18.5)	142 (33.4)	79 (18.7)	89 (21.1)	32 (7.6)
Cost of the medication	99 (23.6)	128 (30.5)	78 (18.6)	82 (19.5)	33 (7.9)
**Pharmacist–patient relationship**					
Pharmacist willingness to answer questions	67 (15.9)	97 (23)	131 (31)	86 (20.4)	39 (9.2)
Respectful care of healthcare providers	64 (15.2)	99 (23.5)	48 (11.4)	151 (35.8)	58 (13.7)
Pharmacists' easy and understandable language	68 (16.1)	94 (22.3)	124 (29.4)	68 (16.1)	66 (15.6)
Spend enough counseling time	46 (10.9)	131 (31)	102 (24.2)	107 (25.4)	34 (8.1)
Getting services in a short waiting time	50 (11.8)	52 (12.3)	136 (32.2)	95 (22.5)	82 (19.4)
**Pharmacist counseling**					
Medication dosage regimen	107 (25.5)	129 (30.7)	65 (15.5)	81 (19.3)	38 (9.0)
Possible side effects	75 (17.9)	92 (21.9)	72 (17.1)	106 (25.2)	75 (17.9)
Medication interactions	102 (24.3)	104 (24.8)	59 (14.0)	101 (24.0)	54 (12.9)
Storage of medicines	93 (22.1)	103 (24.5)	61 (14.5)	84 (20.0)	79 (18.9)
Labeling and dispensing medicines	65 (15.4)	106(25.1)	72 (17.1)	118(28.4)	59 (14)
**Patient satisfaction toward specific pharmacy service**				**Satisfied**	**Unsatisfied**
The structural aspect of the setting				270 (64.3)	150 (35.7)
Medication availability and supply				242 (58.6)	178 (42.4)
Pharmacist–patient relationship				245 (58.3)	175 (41.7)
Pharmacist counseling				258 (61.4)	162 (38.6)
Overall satisfaction				261 (62.1)	159 (37.9)

### Factors associated with pharmacy service psychiatric patient satisfaction among CBHI users

In the current study, gender, age, educational status, number of psychiatric disorder, duration of diseases, availability of prescribed medicine, and waiting time to get hospital service was candidate variables for the final model and entered into multivariable logistics.

In the final model, urban residents [AOR = 2.0; 95% CI (1.25, 3.2), *P* = 0.04], aged between 18–35 and 36–44yrs [AOR = 2.7, 95% CI (1.38, 5.3), *p* = 0.04] and [AOR = 3.3, 95% CI (1.6, 5.7), *p* = 0.001], First second-time institution visitor [AOR = 2.2 (1.15, 4.4), *P* = 0.01], and [AOR = 1.9 (1.13, 3.3), *P* = 0.01], patients who had with two psychiatric disorders [AOR = 1.8, 95% CI (1.07, 3.07), *p* = 0.02], all prescribed medicine available [AOR = 1.5, 95% CI (1.3, 3.43), *p* = 0.02] were significantly associated with the level of psychiatry patient satisfaction on pharmacy services among community-based health insurance users (Table 3).

**Table 3:** Factors associated with pharmacy service psychiatric patient satisfaction among CBHI users hospitals; 2024 (*N* = 420).

**Variables**	**Satisfaction**		**COR (95% CI)**	**AOR (95% CI)**	***p*-value**
	**Satisfied**	**Unsatisfied**			
**Resident**					
Urban	156	111	1.56 (1.02, 2.37	2.0 (1.25, 3.2)	0.04
Rural	105	48	1	1	
**Age**					
18–35 yrs	49	40	1.98 (1.09, 3.6)	2.7 (1.38, 5.30)	0.04
36–44 yrs	57	54	2.3 (1.31, 4.05)	3.03 (1.6, 5.7)	0.001
45–55 yrs	82	35	1.03 (0.58, 1.85)	1.18 (0.62, 2.24)	0.6
>56 yrs	73	30	1	1	
**Education**					
Unable to read	56	60	1.4 (0.83, 2.5)	1.8 (0.97, 3.3)	0.06
Primary (1–8)	71	35	0.8 (0.43, 1.4)	0.7 (0.38, 1.35)	0.3
Secondary (9–12)	70	29	0.6 (0.35, 1.2)	0.5 (0.3, 1.1)	0.09
College and above	55	35	1	1	
**Familiarity to institution**					
First Visit	63	40	1.3 (0.7, 2.2)	2.2 (1.15, 4.4)	0.01
2^nd^ visit	109	75	1.4 (0.87, 2.2)	1.9 (1.13, 3.3)	0.01
More than 3 times	89	44	1	1	
**Psychiatric disorders**					
One	51	28	1.2 (0.65, 2.2)	1.6 (0.77, 3.27)	0.2
Two	123	91	1.6 (1.01, 2.55)	1.8 (1.07, 3.07)	0.02
Three and more	87	40	1	1	
**Duration of disease**					
6–12 months	92	46	0.5 (0.35, 0.98)	0.6 (0.35, 1.05)	0.07
13–24 months	100	54	0.6 (0.39, 1.02)	0.67 (0.39, 1.1)	0.12
>24 months	69	59	1	1	
**Availability of medicine**					
All available	98	50	0.8 (0.4, 1.38)	1.5 (1.3, 3.43)	0.02
Available some	104	72	1.1 (0.66, 1.8)	1.1 (0.6, 1.9)	0.7
Not available	59	37	1	1	
**Waiting time to service**					
< 15 min	99	45	1.6 (0.42, 0.99)	0.67 (0.42, 1.05)	0.08
>15 min	162	114	1	1	

## Discussion

This study's objective was to determine patient satisfaction with pharmacy service among community-based health insurance users. Based on this, overall, we found that more than half (62.1%) of the participants were satisfied with the community-based health insurance (CBHI) member patients during pharmacy service delivery. Over half of the outpatient CBHI users expressed satisfaction or extreme satisfaction with the pharmacy's location about other healthcare services (51.6%) and the availability of prescription pharmaceuticals (51.9%). However, almost half of the participants expressed dissatisfaction and/or significant dissatisfaction with the following: the pharmacy's waiting area's cleanliness (41.9%), the healthcare personnel's respectful care (49.5%), the rate at which the services were provided (41.9%), and the labeling and dispensing of medications (42%).

The degree of patient satisfaction was related to multiple factors. Age, urban resident participants, the number of chronic illnesses, and the availability of prescription medications were all strongly correlated with patients' satisfaction levels in the multiple logistic regressions.

In general, more than 50% of the participants (62.1%) expressed satisfaction with the Pharmacy Service delivery to members of community-based health insurance (CBHI). This result was lower than the study done in Pakistan (92.7%) (36), India (87.28%) (37), Ethiopia (77.6%) (38), and South Africa (72.9%) (39). The possible justification might be due to limited access to mental health services, including long wait times for appointments, perceived lack of empathy from pharmacy professionals, insufficient time spent with patients during appointments, inadequate treatment effectiveness, stigma and discrimination, communication barriers, treatment side effect, inconsistent follow-up or coordination, low insurance coverage, and environmental factors can contribute to lower satisfaction (30, 40).

This result is in line with studies done in Dessie Referral Hospital (61.2%) (41), Amanuel Mental Specialized Hospital (63.3%) (42), Addis Ababa (57%) (43), and India (57%) (44). However, this result was higher than studies done in Dilla (55.4%) (28), Ethiopia, Yekatit 12 referral hospital (47%) (45), and Wolaita Sodo (54.2%) (46). The difference may result from respondents' varying levels of mental health knowledge, improved care, increased awareness and understanding, improved communication between patients and pharmacy professionals, the location of the mental health facility, or the availability of alternative psychological services, better training and education for pharmacy professionals in the study area, healthcare systems increasingly prioritize patient-centered care, and tailoring treatments to individual needs and preferences which can improve overall satisfaction.

Despite the fact that the current study's ratings of satisfaction with the pharmacist's service or communication were consistently higher as compared to previous studies, lower rate of patient satisfaction were noticed with medication availability and supply 178 (42.4%), and pharmacist-patient relationship 175 (41.7%). Even though more than one-third of respondents (37.9%) were dissatisfied with the pharmacy's overall service, they were satisfied with some of the most crucial pharmacist instructions, including pharmacist counseling 258 (61.4%), and overall structural aspect of pharmacy setting 270 (64.3%). According to a national research on the quality of pharmaceutical services (47), patients scored higher on understanding of the dosage (83.2%), frequency (90.8%), and method of administration (94.8%). Additionally, 83.2% and 67.6% of patients, respectively, expressed satisfaction with the administration recommendations and labeling understanding, according to a study done at the Tikur Anbessa specialty hospital (48), which is consistent with this conclusion.

On the structural aspect of the setting, around 177 (41.9%) of psychiatric patients were unsatisfied by waiting area cleanness and comfortability. Furthermore, some patients were dissatisfied with pharmacist counseling including medication side effect 181 (43.1%), counseling on medication interaction 155 (36.9%), and labeling and dispensing of medicines 177 (42.4%). Similarly, results from a published study conducted in Gondar revealed that 62% (31) of participants were dissatisfied with the pharmacist's explanation of the potential side effects of the medicines. The possible reason might be due to lack of a dedicated counseling space in the outpatient psychiatric pharmacy may have contributed to the decreased satisfaction rating in these study areas.

It has been discovered that there is a substantial correlation between residency and patient satisfaction with psychiatric disorders in UOGCSH, FHCSH, and TGCSH public hospitals. In comparison to the reference group of rural residents, participants who were urban residents had higher levels of satisfaction with the community-based health insurance services [AOR = 2.0; 95% CI (1.25, 3.2); *P* = 0.04] and this study finding was supported by a study done in Dilla University Referral Hospital's psychiatry unit (28). The results of this study disagreed with those of studies conducted in Addis Ababa (43) and Dessie (41). The reason might be due to urban areas typically have more pharmacies and healthcare facilities nearby, making it easier for residents to access medications and pharmacy services quickly and conveniently, may offer a wider range of services, such as specialized psychiatric medication management, counseling services, or home delivery options, which can enhance convenience and satisfaction among urban residents, and better healthcare infrastructure, provider-patient relationships, and awareness and education.

In comparison to those 56 years of age and above, participants between the ages of 18–35 and 36–44 were more likely to be satisfied [AOR = 2.7, 95% CI (1.38, 5.3), *p* = 0.04] and [AOR = 3.3, 95% CI (1.6, 5.7), *p* = 0.001], respectively. This finding is consistent with the study done in Pakistan (36). Younger patients in this study may be more accustomed to and at ease with technology, which could be the likely explanation. Better communication with healthcare professionals and a sense of more individualized treatment could result from this, raising satisfaction levels. Higher satisfaction among younger psychiatric patients may also be attributed to the availability of more recent treatment alternatives or therapeutic approaches that better suit the tastes and lifestyles of younger patients. Nevertheless, studies regularly demonstrate that older adult patients are more satisfied than younger ones (49–51). One possibility could be that the older patients' experience and coping mechanisms are what's causing the problem. These traits can help patients accept their condition and the results of their therapy more readily. Higher levels of satisfaction with their overall care may result from this acceptance. Second, compared to younger patients, older people may have different expectations about treatment outcomes and relationships with healthcare providers. Because of their life experiences, they might be more habituated to the healthcare system and have reasonable expectations. Third, constancy in routine: elderly people frequently have more consistency and stability in the management of their mental health conditions. Their sense of control and satisfaction with their care may increase as a result of this consistency. Fourth, longer provider-patient relationships: older patients may have longer-standing relationships with healthcare providers. These relationships can foster trust and understanding, which are important factors in patient satisfaction. Finally, healthcare Engagement: older adults may be more engaged in their healthcare due to their age-related health concerns, leading to better adherence to treatment plans and greater satisfaction with pharmacy service provided.

In the same direction, patients' degree of satisfaction was significantly correlated with having two chronic conditions co-occurring [AOR = 1.8, 95% CI (1.07, 3.07), *p* = 0.02] as opposed to having three or more. This result is consistent with studies done in Boston (51) and Germany (52). Primarily possible reason might be due to the complexity of care: patients with multiple comorbidities often require more complex medical management, including multiple medications, specialist consultations, and frequent follow-ups. This complexity can lead to dissatisfaction among patients who may find it difficult to navigate their care. Moreover, an increased burden: managing multiple health conditions can be physically, emotionally, and financially burdensome for patients. The third one is quality of life issues: comorbidities can significantly impact a patient's quality of life. Ultimately, patients with multiple comorbidities may have higher expectations for their healthcare outcomes and experiences due to the complexity of their conditions. When these expectations are not met, it can lead to dissatisfaction, even if the healthcare provided meets clinical standards.

This study found that the availability of medication in these hospitals was positively associated with patient satisfaction among psychiatrically ill patients with pharmacy service [AOR = 1.5, 95% CI (1.3, 3.43), *p* = 0.02]. This finding is consistent with studies done in St Paulo's Hospital (30). One possible explanation for this could be that patients are satisfied because of the availability of psychiatric drug supplies in hospitals, which decreases cost and extravagance. The second explanation might be due to when medications are readily available; patients can start treatment promptly, which can lead to faster relief of symptoms and improved health outcomes. The final justification could be that if the medication is available in the hospital, it improves adherence, reduces stress and anxiety, improves quality of care perception, and increases trust in the healthcare provider, which leads to their satisfaction with their overall healthcare.

### Implications for clinical practice and policy

In clinical practice, there is a need for enhanced pharmacist-patient interaction to improve communication and patient outcomes. Effective medication management and support for adherence are crucial for achieving better treatment results in psychiatric care. Addressing medication stock-outs is essential to prevent interruptions in therapy and enhance patient satisfaction.

From a policy perspective, integrating pharmacy services into Community-Based Health Insurance (CBHI) is important for ensuring comprehensive mental health care access. Strengthening mental health infrastructure will support better service delivery in psychiatric settings. Additionally, improving the supply chain for psychiatric medications is necessary to maintain consistent availability of essential drugs.

### Suggestions for future research directions

Future research should focus on longitudinal studies to assess how improved pharmacy services impact long-term psychiatric outcomes, especially medication adherence. Comparative studies on CBHI vs. other insurance schemes could reveal its role in enhancing access to psychiatric care. Additionally, research is needed to evaluate pharmacist training effectiveness and examine how socioeconomic and geographic factors influence pharmacy service quality and patient satisfaction, identifying barriers for vulnerable populations.

### Limitation

Because the study only used a quantitative method and a cross-sectional design, it might be difficult to provide more useful information regarding participant perception and the temporal sequence of the associations. Since inpatients, pediatric, and adolescent psychiatric patients were not included in this study, it is challenging to generalize our findings to all psychiatric patients. This is because this study only covered adult patients.

## Conclusion

The community-based health insurance users' satisfaction levels with the pharmacy service were determined to be good in this study. It is noteworthy, however, that factors other than the availability of pharmaceutical services might also have an impact on satisfaction levels. Even with quality pharmacy services, the following extra factors may affect patient satisfaction: care continuity and quality. Furthermore, it was shown that a variety of demographic factors, such as age, residency, and comorbidity, had a significant correlation with patient satisfaction. Medication accessibility was an independent variable that was significantly correlated with patient satisfaction in addition to sociodemographic factors. Therefore, administrators and healthcare professionals should endeavor to improve the hospitals' supply of pharmaceuticals.

## Data Availability

The original contributions presented in the study are included in the article/supplementary material, further inquiries can be directed to the corresponding author.
